# Clozapine and urinary incontinence

**DOI:** 10.1192/j.eurpsy.2022.731

**Published:** 2022-09-01

**Authors:** A. Osca Oliver, M. Palomo Monge, M.F. Tascón Guerra, M. Pérez Fominaya, M.V. López Rodrigo

**Affiliations:** 1 Hospital Nuestra Señora del Prado, Psiquiatría, Talavera de la Reina, Spain; 2 Hospital Nuestra Señora del Prado, Psiquiatria, Talavera de la Reina, Spain

**Keywords:** urinary incontinence, adverse effects, clozapine, schizophrénia

## Abstract

**Introduction:**

Clozapine is an atypical antipsychotic, which has been shown to have superior efficacy in the remission of positive and negative symptoms in patients with treatment-resistant schizophrenia, compared to other antipsychotics. However, its benefits have been limited by the lethal adverse effects that this drug can cause, the most common, agranulocytosis. Other less serious and more common adverse effects are sedation, hypersalivation, hypo and hypertension, weight gain and urinary incontinence. It is estimated that approximately 1% of patients treated with Clozapine suffer from urinary incontinence. Data that varies from 0.3% to 42% in the articles reviewed, being undervalued in some of these for many reasons; it is stigmatizing and the patient is ashamed to express it.

**Objectives:**

The objective of this study is to assess the percentage of urinary incontinence in patients in treatment with clozapine, taking into account the presence or absence of this side effect as the main variable, and the diagnosis, gender and dose of clozapine as secondary variables.

**Methods:**

A retrospective observational study was carried out in which 40 patients belonging to the Adult Mental Health area of the Nuestra Señora del Prado Hospital, were collected, all of them diagnosed with some type of psychotic disorder and undergoing treatment with Clozapine.

**Results:**

Of the total of patients studied, 25% presented urinary incontinence as an adverse effect, and of these, 60% were with doses equal to or greater than 400 mg of Clozapine.

**Conclusions:**

We must be careful and bear this side effect in mind in all patients taking Clozapine.

**Disclosure:**

No significant relationships.

